# Research on the Factors Affecting the Adoption of Smart Aged-Care Products by the Aged in China: Extension Based on UTAUT Model

**DOI:** 10.3390/bs13030277

**Published:** 2023-03-21

**Authors:** Xiang Wang, Chang-Franw Lee, Jiabei Jiang, Genlei Zhang, Zhong Wei

**Affiliations:** 1 Graduate School of Design, National Yunlin University of Science and Technology, Yunlin 64002, Taiwan; 2 Pujiang Institute, Nanjing Tech University, Nanjing 211200, China; 3 The Future Laboratory, Tsinghua University, Beijing 100000, China; 4 Faculty of Social Sciences and Liberal Arts, UCSI University, Kuala Lumpur 56000, Malaysia

**Keywords:** the aged, smart aged-care products, user behavior, unified theory of acceptance and use of technology, perceived cost, perceived risk, structural equation model

## Abstract

With the rapid development of network technology and smart technology, smart aged-care products are becoming increasingly valued for their ability to help the aged actively cope with the challenges of aging. However, seniors face challenges in using smart aged-care products for many reasons, which reduces their willingness to adopt them. As a result, the sustainable development of smart aged-care products is constrained. This study combined the unified theory of technology acceptance and use, perceived risk theory and perceived cost theory, and reconstructed a research model that investigated the adoption of smart aged-care products by the elderly in China. Questionnaires were given to older Chinese adults in this study, and 386 valuable responses were received. The findings of the structural equation model (SEM) analysis are as follows: (1) performance expectancy, effort expectancy, and social influence were positively related to the behavioral intention of seniors to use smart aged-care products; (2) perceived cost and perceived risk were negatively related to the behavioral intention of seniors to use smart aged-care products; (3) perceived risk indirectly affected use behavior through behavioral intentions; (4) facilitating conditions did not have a significant impact on the use behavior of seniors in adopting smart aged-care products. Based on the empirical results, this study sought to improve the use behavior of the aged in relation to the adoption of smart aged-care products, and provided suggestions to improve the overall service quality and sustainability of those products.

## 1. Introduction

### 1.1. Research Background

China is currently facing the enormous challenge of rapid population aging on a large scale, with people becoming elderly without the necessary financial resources. A successful response to aging depends to a large extent on the ability to improve and maintain the health of the aged; that is, successful aging is healthy aging [1]. Smart aged-care service technology has the potential to help the aged in monitoring and maintaining their health, and in managing health conditions and diseases [2]. Additionally, the use of smart aged-care products is one of the solutions to the problem aged-care [3].

**Figure 1:**
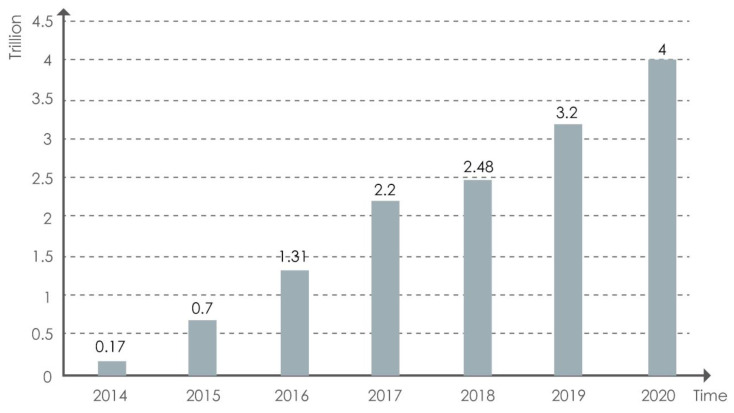
Market scale of smart aged-care industry in China during 2014–2020 [6].

Based on data from the National Bureau of Statistics of China, the population of people over 65 years old is constantly rising, and comprises 12.64% of the total population of the Chinese mainland as of 2020, which growth is speeding up. According to the UN’s statistics, people over 65 years old in China comprise 7% of the total population, indicating the nation is becoming an aging society [4]. The proportion of those over 65 years old in China is well above 7%, indicating its nearness to becoming an advanced aging society [5]. As the aging of China’s population further intensifies, it will gradually become a deeply aged society, and the demand placed on the smart aged-care industry is continuing to rise. In 2019, the size of China’s smart aged-care industry was nearly CNY 3.2 trillion, with a compound growth rate of more than 18% over the past three years; the industry was expected to reach CNY 4 trillion by 2020 (as shown in Figure 1) [6]. Continuous breakthroughs will also drive the continuous development of the smart aged-care product industry.

It was found that the aged are worried about smart technology and discouraged from adopting it [7]. According to the 47th Statistical Report on the Development Status of the Internet in China, released by the China Internet Network Information Center (CNNIC), as of December 2020, the proportion of the netizen group in China aged 60 and above was only 11.2% [8]. These data partially reflect the low familiarity of the elderly with modern technology products, and suggest the majority may encounter barriers to using smart products. However, the effectiveness of smart elderly products has been demonstrated by many scholars. Pilotto et al. [9] studied 223 Alzheimer’s patients who used information technology, and showed that this technology was effective in improving their quality of life, quality of service, and safety. Chan et al. [10] pointed out that intelligent technologies can prevent behaviors that endanger health through health warnings. The results of the study by Faucounau et al. [11] show that intelligent robotics can play a useful role in social interaction, psychological guidance, educational learning, cognitive enhancement, and intelligent monitoring for the elderly.

### 1.2. Research Purpose

The popularity and adoption of smart aged-care products in China are not only related to the development of the technology and services but are closely related to the acceptance of these products by the elderly. In order to better understand and predict the adoption behavior of the aged towards smart aged-care products, it is important to explore the influencing factors. Currently, foreign research on smart elderly products is relatively mature, mainly focusing on research on product system development and smart homes, with relatively little research on the demand for smart elderly products [12]. Chinese research is still in its infancy, mostly focusing on theoretical constructs, and it does not extend to actual demand research [13].

This study combined the unified theory of acceptance and use of technology, the theory of perceived cos, and the theory of perceived risk and constructed a research model to analyze the factors influencing the adoption of smart aged-care products by the aged. It will fill a gap in the research on this issue in China and provide recommendations for the development of the smart aged-care products industry as well as theoretical guidance for government departments and service providers, while also providing suggestions to ensure the adoption of these products by the aged.

## 2. Literature Research

### 2.1. Smart Aged-Care Products

Lacking a rigorous definition in the academic world, and using the previous literature [14,15,16], we define smart aged-care products as products that incorporate advanced modern technology, as well as new smart hardware products and smart aged service information platforms, which are able to help the aged actively cope with aging. These technologies can fit into two main categories, namely, smart service platform systems for the aged, and smart service terminals for the aged. Among these, smart service platform systems for the aged refer to products that rely on information technology to perceive, transmit, publish, and integrate the service needs of the aged, and can facilitate the communication between medical care, services, families, and individuals to meet the diversified and multifaceted needs of the aged. Smart aged-care terminals are smart devices that incorporate advanced technology, such as Robot Doctors, smart nursing robots, companion robots, and other types of service robots.

### 2.2. Research Status of Smart Aged-Care Products

Recent studies on smart aged-care products mainly focus on two aspects: technical innovation and product development, such as Ref. [17], which concerns the application of smart material sensors and soft electronics in wearable healthcare devices, and Ref. [18], which concerns physiotherapy programs aided by virtual reality (VR) solutions; and service platforms and service modes, such as Ref. [19] on the improvement of countermeasures incorporated into digital health service platforms based on empirical research, and Ref. [20] on the construction of smart pension cloud platforms based on big data technology. To date, negligible advancements have been in China in assessing the willingness of the aged to use smart technology products and the corresponding influencing factors, and these achievements have mainly been made via qualitative research. Few scholars have performed quantitative analyses on this issue, and even fewer results have been derived specifically concerning the willingness of the aged to use smart technology products and its corresponding influencing factors. Some scholars have studied the factors influencing the preferences and behaviors of the aged via the unified theory of acceptance and use of technology and the diffusion of innovation theory. However, these fail to consider the influences of costs and risk factors on the use behaviors of the aged in China, based on their actual conditions. As China is a developing country, a great number of its aged citizens are of relatively low education and income level. In light of this and the actual conditions of the aged in China, this study specifically addresses the influences of perceived risk and perceived cost factors on the use behavior of the aged, and thus extends the UTAUT model.

### 2.3. UTAUT Model

**Figure 2:**
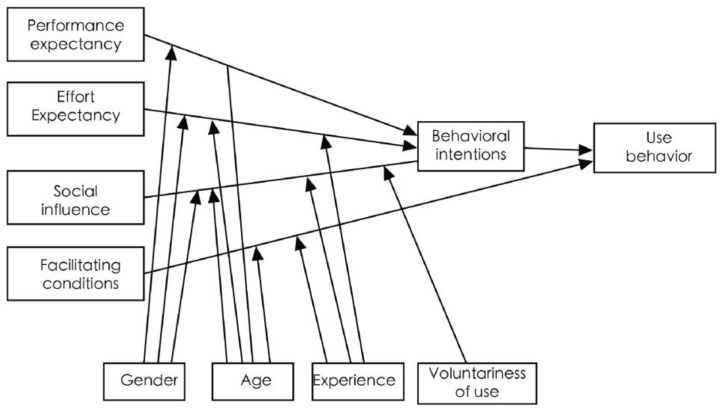
UTAUT model.

Venkatesh et al. [21], Davis [22], and others have proposed an integrated model of technology acceptance and use (UTAUT) that can more accurately depict the actual situation by thoroughly considering the theory of reasoned behavior, the technology acceptance model, the computer use model, the theory of planned behavior, innovation diffusion theory, social cognition theory, the TAM and TPB models, and the motivation model. Our in-depth study of the UTAUT model (see Figure 2) effectively integrates the strengths and characteristics of these eight models to yield an integrated model of technology acceptance and use (UTAUT) that can more accurately predict the actual situation. The UTAUT model integrates the main considerations of the eight models into four core variables—performance expectations, effort expectations, social influence, and contributing factors—and four moderating variables—gender, age, experience, and voluntariness-related constructs and explanations. 

Performance expectation (PE) refers to the increase in performance that the user anticipates the technology or product will bring. This variable is used to measure the extent to which the technology and product will be helpful to the user. Effort expectation (EE) refers to the ease with which the user expects they will be able to accept the technology or product. This variable is used to measure the degree of effort required by the individual in accepting the technology or product. Social Influence (SI) is defined as the impact of the adoption of this technology or product on other people who are perceived by the user as having high social status or as being important. This variable is used to measure the extent to which the user is influenced by social groups. Facilitating conditions (FC) refers to the degree to which users believe that the existing technology or product will be supported by social groups and further technological developments. This variable mainly measures the perceived convenience of the available support for using the technology or product.

The UTAUT model has a wide range of applications in the field of smart aging [12,23,24,25,26,27]. Since smart aged-care products are a relatively new type of product, an integrated technology acceptance model can help us explore the key factors affecting the adoption of smart aged-care products by the aged. This paper is focused on the willingness to adopt, and therefore excludes the two control variables of experience of use and voluntariness from the model. Related studies have shown that there is no significant difference as regards the gender and age of seniors in adopting smart aged-care products. Therefore, these two control variables were not included in the model of this study.

### 2.4. Perceived Cost

Perceived cost is the sum of the expenses that customers feel they have had to pay during the actual consumption process, and compiles costs related to time, money, physical effort, energy, and psychological effort [28], not just the actual price paid by the customers. Perceived cost theory has been widely applied in the field of smart aged-care [29,30,31]. The high initial costs associated with any new technology, product, or service are often seen as a major barrier to its acceptance [32,33,34]. As an emerging type of technology and product, the adoption of smart aged-care products by the aged will necessitate more time spent in learning, more money spent in purchasing, more effort expended in use, etc. The excessive costs will reduce the behavioral intention of the aged to use smart aged-care products. Therefore, this study will use perceived cost theory to explore the effects of perceived costs on the adoption of smart aged-care products by the aged.

### 2.5. Perceived Risk

Perceived risk theory was first conceptualized by Bauer, a professor at Harvard University. Bauer [35] experimentally confirmed that people’s behavioral outcomes cannot be accurately predicted before they begin to act, and the outcomes resulting from people’s behavior can be either good or bad. However, people anticipate the riskiness of behavioral outcomes before the act begins, the product of which is referred to as perceived risk. Perceived risk is defined as the user’s ultimate expectation of an unpleasant outcome, or of an end result that is detrimental to the individual. Taylor [36] argued that users’ decisions regarding adoption are related to their perception of risk. Perceived risk theory has a wide range of applications in the field of smart aged-care products [37,38,39,40]. Perceived risk is related to the existence of uncertainty, and the aged often face many uncertainties when adopting smart aged-care products. This study will use perceived risk to explore its impact on the adoption of smart aged-care products by the aged.

## 3. Research Hypothesis and Methodology

### 3.1. Research Hypothesis

Based on a study of the literature, we propose several research hypotheses regarding the factors that influence the adoption of smart aged-care products by the aged.

#### 3.1.1. Relationship between Performance Expectations and Behavioral Intentions

Performance expectations are a rich variable based on perceived usefulness, and their significant effect on user adoption has been confirmed by several studies [41,42,43,44,45,46]. For example, Prasetyo et al. [41] confirmed that performance expectations had a positive effect on behavioral intentions to receive medical-educational e-learning and found that performance expectations had the greatest effect on behavioral intentions; Oliveira et al. [43] confirmed in their empirical study that performance expectations had a positive relationship with behavioral intentions. Performance expectancy implies that seniors expect to benefit from the use of smart aged-care products. Therefore, Hypothesis 1 was proposed.

**Hypothesis** **1.** *The performance expectation of the aged is positively related to their behavioral intention to adopt smart aged-care products.*

#### 3.1.2. Relationship between Effort Expectation and Behavioral Intention

Effort expectancy has a similar impact on perceived ease of use in the TAM model and refers to whether the user perceives the technology or product as easy to incorporate into use. Several empirical studies have demonstrated that ease of use metrics have a significant impact on users’ behavioral intention [23,27,47,48]. For example, Mao and Li [27] demonstrated through empirical methods that effort expectancy had a positive influence on behavioral intention. Cimperman, M et al. [23] confirmed that effort expectancy directly influenced behavioral intention to use home telehealth services. Therefore, Hypothesis 2 is proposed.

**Hypothesis** **2.** *The effort expectation of the aged is positively related to their behavioral intention to adopt smart aged-care products.*

#### 3.1.3. Relationship between Social Influence and Behavioral Intention

Venkatesh et al. [21] stated that social influence positively affects individuals’ intention to adopt under the UTAUT. Studies by Hsu and Peng [49], Mabkhot [50], Nysveen et al. [51], and Hoque and Sorwar [12] have shown users influenced by their surroundings and social environment when using services, whose intention to use was enhanced when those in their immediate surroundings were already using, or when the social climate encouraged it. This study thus explores whether the intention to adopt among seniors is positively influenced by the surrounding environment. Therefore, Hypothesis 3 is proposed.

**Hypothesis** **3.** *Social influence is positively related to behavioral intention*.

#### 3.1.4. Relationship between Facilitating Conditions and Use Behavior

Yang et al. [52] empirically demonstrated that facilitating conditions positively influenced the smartphone use behavior of older adults. Wang et al. [53] empirically demonstrated that facilitating conditions positively influenced consumers’ behavioral intention to use wearable healthcare devices. Cimperman, M et al. [23] empirically demonstrated that facilitating conditions directly influenced older users’ behavioral intention to use home telehealth services. In this study, facilitating conditions are described as the ability of the senior to use the support resources, and the conditions associated with using the smart aged-care product. When these necessary resources are available, seniors display use behavior related to smart aged-care products. Therefore, Hypothesis 4 is proposed.

**Hypothesis** **4.** *Facilitating conditions are positively related to use behavior.*

#### 3.1.5. Relationship between Behavioral Intention and Use Behavior

Behavioral intention refers to a user’s tendency to adopt a certain behavior, and as theorized by UTAUT, behavioral intention has a positive impact on user behavior. Oliveira et al. [43] empirically confirmed that behavioral intention to use m-banking had a positive impact on user adoption. Prasetyo et al. [41] confirmed in their study that behavioral intention has a positive impact on the use of an e-learning platform. In the study by Zhang M. et al. [54], it was confirmed that behavioral intention positively influenced use behavior. This study explored the behavioral intention of the aged to adopt smart aged-care products, finding a positive impact on use behavior. Therefore, Hypothesis 5 is proposed.

**Hypothesis** **5.** *Behavioral intention is positively related to use behavior*.

#### 3.1.6. Relationship between Perceived Cost and Behavioral Intention

Zainab et al. [55] found that perceived cost had a significant impact on adoption intention related to e-training. Gupta et al. [56] found that perceived cost had a negative impact on behavioral intention related to knowledge sharing. Zhang and Gao [31] concluded that perceived cost had a negative impact on older adults’ use of online health information services, in terms of the negative use behavioral intentions of avoidance and withdrawal. This study explored the effect of perceived cost on behavioral intention among seniors. Therefore, Hypothesis 6 is proposed.

**Hypothesis** **6.** *Perceived cost is negatively related to behavioral intention*.

#### 3.1.7. Relationship between Perceived Risk and Behavioral Intention and Use Behavior

In a study by Wang [39], it was confirmed that the risks perceived by the aged regarding the use of smart aged-care services affect the intention to use such services. A study by Khan et al. [57] found that perceived risk negatively influences users’ adoption of new technology. A study by Ku and Hsieh [40] found that perceived risk was a key factor influencing the acceptance of cloud-based healthcare services among the elderly in Taiwan. In this study, we assume that seniors face many risks and challenges related to using smart aged-care products. For that reason, we will explore whether perceived risk affects adoption behavioral intention and use behavior. Therefore, we propose Hypothesis 7—that perceived risk is negatively related to behavioral intention—and Hypothesis 8—that perceived risk is negatively related to use behavior.

### 3.2. Research Structure

**Figure 3:**
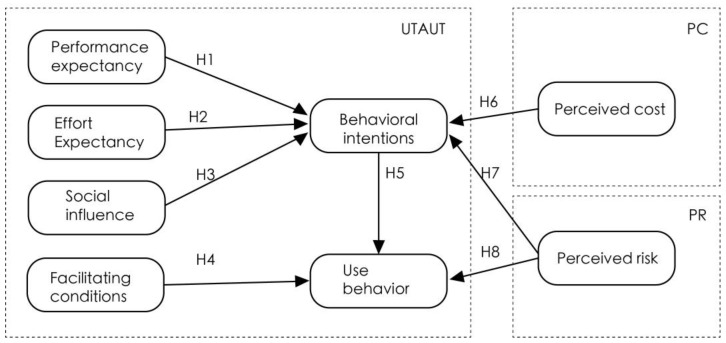
Research model.

We constructed the research model of this paper by integrating the integrated technology acceptance model, perceived cost theory and risk perception theory, based on the previous research undertaken by its authors, as shown in Figure 3.

### 3.3. Definition and Measurement of Variables

We first constructed the research model and hypotheses (as indicated in Figure 3). Eight factors ultimately affecting behavioral intentions have been determined, including performance expectations, effort expectations, social influence, facilitating conditions, perceived costs, perceived risks, behavioral intentions, and use behaviors. To ensure the reliability and validity of the variables, on the basis of the relevant national and international literature, this study has established 33 questions on a rating scale; that is, one question corresponding to each dimension in the questionnaire (as shown in Table 1).

## 4. Data and Methods

### 4.1. Questionnaire Survey

This study employed a questionnaire survey for the purpose of data collection, with 37 questions in total split across 3 parts. The first part concerned the definition and introduction of smart aged-care products. Products such as “Xiaomi Smart bracelet”, “Yuyue Smart blood glucose detector”, and “Folca Smart medicine box”, which are most commonly used in China, were listed in the questionnaire for illustrative purposes. The second part concerned the basic information of the aged, including gender, age, education level, monthly income, etc. (four questions in total). The third part sought to measure the willingness of the aged to use smart aged-care products with 33 questions in total; that is, 1 item corresponding to each dimension.

This study employed a questionnaire survey for data collection, and we performed pretesting to determine the questionnaire’s reliability prior to the formal commencement. A 7-point Likert scale was used in the pre-test questionnaire, which was conducted from 14 July to 23 July 2022 with 50 copies distributed to aged users of smart devices in nursing homes in Jiangning Disitrct and Gulou District in Nanjing. We analyzed the reliability values and the items of the pre-test questionnaire, and we eliminated undesirable items to ensure more accurate research results and enhance sample reliability and distinction capacity.

Formal questionnaires were distributed to smart aged-care products users over 60 years old in places with relatively high numbers of aged people, such as smart aged-care communities, and in the aged activity centers and nursing homes in Jiangsu Province and the surrounding areas, from 14 August to 10 September 2022. A total of 450 copies were distributed and 386 valid questionnaires were received, with a validity rate of 85.78%.

This study used the Cronbach’s α of statistical software Spss 22.0(IBM Corp., Armonk, NY, USA) to review the scale’s reliability. As shown in Table 2, the values of Cronbach’s α are 0.916, 0.909, 0.907, 0.906, 0.898, 0.895, 0.874, and 0.935 for performance expectation, effort expectation, social influence, facilitating conditions, behavioral intention, use behavior, perceived cost, and perceived risk, respectively, all of which are higher than 0.7, indicating good scale reliability (as shown in Table 2). 

### 4.2. Sample and Data Collection

(1) As shown by Table 3, the proportion of females using smart aged-care products is 51.55%, which is essentially equal to the proportion of males—48.45%. This indirectly reflects that the use of smart aged-care products does not differ by gender. (2) The proportions of those who were 60–65 years old, 66–70 years old, 71–75 years old, 76–80 years old, and over 80 years old were 16.84%, 25.13%, 27.98%, 22.80%, and 7.25%, respectively; those over 80 years old were placed in the hyper-aged group, comprising 7.25% of the users—this was a smaller group than the other age groups, into which the users were relatively equally distributed. (3) In terms of educational background, the proportions of those with senior high school education and under, junior college education, undergraduate education, and Master’s education and above were 75.65%, 6.48%, 12.95%, and 4.92%, respectively; in relation to the specific conditions of China, those with senior high school education and under constituted 75.65% of the sample, meaning this variable could not be used to assess the effect of educational background on the use of smart aged-care products. (4) In terms of monthly income, the proportions of those earning below CNY 2000, CNY 2001–3500, CNY 3501–5000, and over CNY 5000 were 8.29%, 20.21%, 32.38%, and 39.12%, respectively. We can see that with higher monthly incomes, the proportion of people using smart aged-care products increases. This also verifies the impact of perceived cost on the behavioral willingness to adopt smart care products. The samples collected in this study were relatively evenly distributed across the range of demographic variables, which fulfilled our expectation.

### 4.3. Validity Analysis

#### 4.3.1. KMO and Bartlett Tests

Validity generally refers to the accuracy of the test results and is normally measured by the difference between the test results and the test objective. The construct validity here was derived via SPSS factor analysis to verify whether the scale could meet the demands of the research objective. KMO and Bartlett’s test of sphericity were adopted to verify the relevant variables, and the results are shown in Table 4. As regards the KMO test, the values given should fall between 0 and 1; the closer the value is to 1, the stronger the correlation between the variables is. Normally, only statistics with values above 0.7 are taken as fit for factor analysis, while those below 0.5 are not. The KMO value of the results in the table is larger than 0.9, and the *p* value of the Bartlett test of sphericity is 0, indicating that the preliminary validity test yielded good results.

#### 4.3.2. Exploratory Factor Analysis

Exploratory factor analysis was applied here to the 8 structures of the hypothesis model, which abstracts 8 factors with eigenvalues larger than 1. The factor loading of each item was larger than 0.6 and the cumulative percentage of variance explained was 77.993%, indicating the high validity of the abstracted common factors. As can be seen from Table 5, a total of 8 common factors were extracted, which showed consistent pre-dimensionality, further indicating good validity.

### 4.4. Measurement Model

#### 4.4.1. Convergent Validity

For the structural equation model analysis, we used AMOS 17.0, a provably reliable modeling software for structural equations with wide application in a great number of studies. According to Anderson and Gerbing [64], data analysis can be divided into two stages. The first stage involves establishing the measurement model, and applying maximum likelihood estimation to the estimated parameters, including factor loading, reliability, convergent validity, and discriminative validity. The second stage involves the research conducted by Hair et al. [65], Nunnally and Bernstein [66], Fornell and Larcker [67], Chin [68], and Hooper et al. [69] on convergent validity. As shown in Table 6, the standardized factor loadings for the entire variable are larger than 0.7, indicating that each variable under observation will be significantly helpful in explaining its latent variable. It should be noted that the value of CR is larger than 0.8, and thus higher than the standard of 0.7. As such, the observed variables for each dimension can effectively explain the respective dimension. All the values of AVE derived here are above the standard 0.6, indicating the good convergent validity of the scale.

#### 4.4.2. Discriminant Validity

The approach of Fornell and Larker [67] has been adopted for the study of the validity of the discriminant. The sample data used in the model show good discriminant validity if all the AVE square roots of each dimension are larger than the absolute values of the correlation coefficients between each variable. The results show that the values shown on the diagonal are larger than others. Consequently, all the structures here are interpreted to be valid (see Table 7), and each aspect of the study is of high discriminant validity. 

### 4.5. Structural Model Analysis

#### 4.5.1. Model Fit Criteria

The model fitting index is here used to test the existing models, inspect the fitting degree of the models to the collected variable data, and compare the degree of coincidence between the prediction results and the actual situation. Based on research by Jackson et al. [70], Kline [71], Whittaker [72], Hu and Bentler [73], and other scholars, we selected a number of indexes (MLχ2, DF, χ2/DF, RMSEA, SRMR, TLI, CFI, GFI, and IFI) to evaluate the structure model’s fit, and the results are shown in Table 8, which were derived after parameter measurement was applied to the model and the hypothesis. It can be seen from the data in the table that the actual measured data fit well. Therefore, no further modifications to the model were required, and path analysis could be conducted.

#### 4.5.2. Path Analysis

As shown in Table 9, the performance expectation (R = 0.28, *p* < 0.001) has a positive correlation with the behavioral intention of the elderly to use smart aged-care products; that is, H1 is supported. Effort expectation (R = 0.27, *p* < 0.001) has a positive correlation with the behavioral intention of the elderly to use smart aged-care products; that is, H2 is supported. Social impact (SI) (R = 0.20, *p* < 0.001) has a positive correlation with the behavioral intention of the elderly to use smart aged-care products; that is, H3 is supported. Facilitation conditions (R = 0.018, *p* = 0.761) have no significant impact on the use behavior of smart aged-care products for the elderly; that is, H4 is not supported. Behavior intention (BI) (R = 0.86, *p* < 0.001) positively affects the use behavior; that is, H5 is supported. Perceived cost (R = −0.25, *p* < 0.001) has a negative correlation with the behavioral intention of the elderly to use smart aged-care products; that is, H6 is supported. Perceived risk (R = −0.25, *p* < 0.001) has a negative correlation with the behavioral intention of the elderly to use smart aged-care products; that is, H7 is supported. Perceived risk (R = 0.10, *p* = 0.222) has no significant impact on the use behavior of intelligent elderly care products for the elderly; that is, H8 is not supported.

### 4.6. Hypothesis Explanation

**Figure 4:**
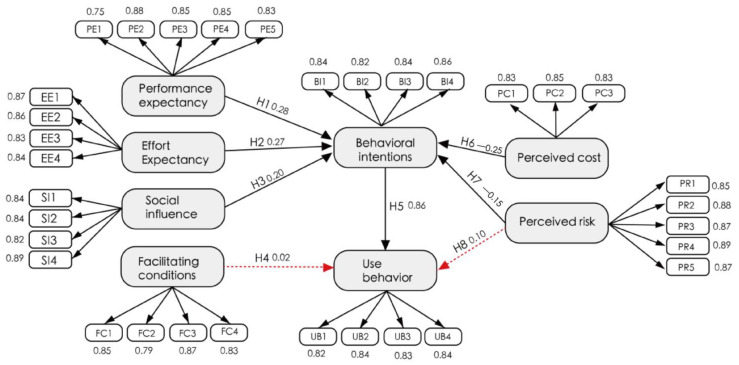
Research Model Diagram.

In Figure 4, the regression coefficients of the SEM model used in this study are shown. A higher coefficient means that the independent variable plays a more important role that the dependent variable. The assumptions of H1, H2, H3, H5, H6 and H7 are valid, while the assumptions of H4 and H8 are not.

### 4.7. Results and Discussion

This study constructed an impact model of the willingness of the aged to adopt smart aged-care products, based on the unified theory of acceptance and use of technology, perceived cost theory, and perceived risk theory. The results of the empirical analysis of the UTAUT extended model show that performance expectancy, effort expectancy, social influence, behavioral intention, perceived cost, and perceived risk influenced the acceptance of smart aged-care products by seniors. As regards impact effects, that of behavioral intention was the largest at 0.86, while that of performance expectation was 0.28, the effort expectation impact effect was 0.27, the perceived cost impact effect was 0.25 (negative correlation), the social impact effect was 0.20, and the perceived risk impact effect was 0.15 (negative correlation).

(1) Performance expectations have a positive effect on behavioral intentions, which result is consistent with previous findings [32,33,34,35,36]. The performance expectation factor has the largest effect other than behavioral intention, and Sun et al. [74] showed that the higher the performance expectation, the stronger the willingness to use, which verifies the UTAUT model. This indicates that seniors will be willing to adopt smart aged-care products when they think they can improve the services available to them. Therefore, considering seniors’ demands, the service providers should introduce personalized services in addition to the basic functions, targeted to improve the use expectations of the aged [39].
(2) Effort expectancy has a positive effect on behavioral intention, which finding is consistent with those of previous studies [23,27,47,48], indicating that the aged are more willing to adopt smart aged-care products when they are perceived as easy to use. It can be seen that the ease of use of smart aged-care products is a key concern for service providers, who should provide services that are as convenient and easy-to-use as possible. In view of the problem that the aged do not have sufficient skills to use smart aged-care products and that they are not easy to use, it is necessary to increase the diversification of smart aged-care products and services, and thus develop smart aged-care products that are more suitable for use.
(3) Social influence has a positive impact on behavioral intention, which result is consistent with previous findings [12,21,50,51], suggesting that when the aged individual perceives a higher level of acceptance of smart aged-care products among those around them, their intention to adopt will rise. At present, the aged generally have a low level of understanding of smart aged-care products, and few have actually used and experienced them [75]. Chen et al. [76] argued that there are few channels for smart aged-care product use, leading to a relatively low degree of willingness to use and a lower frequency of use behavior amongst the aged. Society has not yet developed sufficient motivation to encourage aged groups to accept and use advanced technology. This means that service providers should pay more attention to their own approaches to publicity, as an increase in social recognition can enhance the trust of users and thus improve their willingness to use.
(4) There is no significant effect of facilitating conditions on the use behavior of smart aged-care products among the elderly. This result is inconsistent with those of previous studies [23,52,53,77], and indicates that China has not yet developed the necessary resources to support the use of smart aged-care products amongst the elderly. (1) The current Chinese government and other concerned organizations seek to assist the aged mainly via economic benefits and donations, which do not contribute to their use of smart aged-care products; (2) instead of individually targeted services, the care-related actions of family members, communities and volunteers are more focused on short-period companionship and care, and thus fail to develop the necessary recourses that will support the systematic use of smart aged-care products; (3) instead of developing follow-up support services, such enterprises prefer to focus on sales in relation to short-term interests. As they age, people undergo physiological and psychological changes, which cause their learning ability and attention to gradually decline [78]. At the same time, the aged perceive internet technologies as high-tech products that require a whole new body of knowledge to navigate. Therefore, technophobia is common [79]. This requires the government, market, community, and family members to develop better resources, enhance technical support and usage knowledge, and develop more inclusive products. This will help in offering the timely assistance required when aged people use smart aged-care products. In relation to this, the government could encourage family members to bear more responsibilities in relation to the smart education of the aged. Both communities and public service organization should provide the aged with more targeted services related to education on the use of smart aged-care products; the service organizations concerned may even prepare some accessible literature instructing on the use of smart aged-care products, thus making information resources and assistance accessible when necessary. Furthermore, enterprises must establish complete service systems for smart aged-care products, and provide the aged users with training, maintenance, and product-upgrading services, thus enhancing the healthcare services that use smart aged-care products.
(5) Behavioral intention has the greatest positive influence on use behavior, which is consistent with previous findings [32,44]. It has been indicated that positive behavioral intention can promote use behavior among the aged. Gamma et al. [80] concluded that behavioral intention and use behavior are highly correlated. Behavioral intention is constructed from the senior user’s own experience, cognitive abilities, and needs to be met by the care services. Therefore, in order to improve the use behavior of seniors, it is necessary to improve their behavioral intention, increase their relevant experience, improve their cognitive abilities in relation to the use of smart aged-care products, and meet their care needs in a targeted manner in order to help develop a positive attitude toward smart aged-care products.
(6) Perceived cost has a negative impact on behavioral intention, which result is consistent with previous findings [31,34,55,56]. It has been indicated that factors such as excessive monetary, learning and time costs significantly reduce the behavioral intention of the aged to use smart aged-care products. Smart aged-care products, as information technology-based products, are more expensive than typical products, and policy and financial support from the government is required in order to reduce the associated costs. At the same time, service providers are required to improve the user-friendliness of their smart aged-care products in order to reduce the associated learning and time costs.
(7) Perceived risk is negatively related to the behavioral intention of the aged to adopt smart aged-care products, which result is consistent with previous findings [36,39]. However, perceived risk is not significantly related to use behavior, which is inconsistent with previous findings [40,57]. It has been indicated that the risks perceived by the aged have a negative impact on their behavioral intention to adopt smart aged-care products, and also indirectly influence use behavior. This can be explained with reference to the dimension of use behavior and the measurement items selected in this study. Related to the rapid growth and application of internet technology, the use of smart aged-care products is emerging as a trend that is making later life much safer, more convenient, and more comfortable. However, the aged tend to be concerned about risks such as the security of use and privacy breaches when using smart aged-care products, and these risks influence their related behavioral intentions. The strengthening of security assurances related to smart aged-care products will help to establish a sense of trust and safety amongst the aged in relation to smart aged-care products, thus enhancing their use behavior. The government is duty-bound to set up security systems concerning at smart aged-care products, form security supervision mechanisms in collaboration with judicial and public safety departments for the joint consideration of network information security issues and ensure both the personal and financial security of the aged during their use of smart aged-care products.

## 5. Conclusions

### 5.1. Theoretical Contribution

Research on this topic in China is still in its infancy, and previous studies on smart aged-care products have mostly focused on the design, development, and application of specific models of smart products and their effects, with little focus on user behavior. This study enhances the research on user behavior in relation to smart aged-care products in China and enriches our understanding of smart aged-care and adoption intention. This study focuses on the elderly in China. The demand for healthy aged-care is strong among the elderly, but the adoption rate of smart aged-care products is low due technology acceptance, cost, and risk factors. Therefore, this study has adopted a new perspective focusing on the influence of these factors and has constructed a research model addressing the adoption of smart aged-care products by Chinese seniors based on the UTAUT model combined with perceived risk and perceived cost theories. The model provides a comprehensive means of understanding the adoption of smart aged-care products.

This study has derived some valuable conclusions through its empirical research analysis: (1) performance expectation, effort expectation, and social influence are positively related to the behavioral intention of the aged to adopt smart aged-care products; (2) behavioral intention is positively related to use behavior and shows the greatest effect of all factors studied; (3) we found no significant effect of facilitating conditions on seniors’ use of smart aged-care products, which yields a more accurate perception of the resources that must be developed to ensure smart aged-care product support; (4) perceived cost is negatively related to behavioral intention; in this study, perceived cost is defined as the time, money, and effort required in order for the aged to adopt smart aged-care products; these factors significantly affect willingness to adopt since China is still a developing country with low income amongst the aged; (5) many studies have shown that perceived risk is an inhibiting factor that hinders individuals’ intention to adopt. This study confirms that perceived risk has a negative effect on the behavioral intention of the aged to adopt smart aged-care products, and this indirectly (but not directly) affects use behavior. These findings have important theoretical implications and will help in proposing solid recommendations.

### 5.2. Practical Contribution

As the issue of the aging population in China is becoming increasingly serious, smart aged-care products can provide timely, efficient, and convenient care services for the aged, which will help to significantly improve and maintain the health of the aged. They thus represent one of the main paths to achieving healthy aging. If the user behavior of smart aged-care products amongst the aged is to be improved, service providers must strengthen the positive influencing factors and address the negative influencing factors. For example, they must (1) improve the performance expectation, effort expectation, social impact, and behavioral intention of smart aged-care products, and pay attention to the usefulness, ease of use, and public promotion of smart aged-care products; (2) reduce the learning time, purchasing, and use effort costs related to smart aged-care products; (3) address concerns about the risks related to using smart aged-care products, and enhance the trust of the aged. The results of this study have important practical implications that will help providers improve the overall quality of smart aged-care products and services.

The practical value of this study is in the fact that it offers an objective view of the actual needs of the aged in China in terms of smart aged-care products and the roles they play. It also provides a reference for other similar studies on the use behavior related to smart aged-care products, which will be conducive to improving the adoption level and health literacy of the aged.

## 6. Limitations and Future Prospects

Firstly, the data collected in this study are limited to the aged in Jiangsu Province and the surrounding areas. Due to the differences in economic level and other corresponding factors between provinces, the use of smart service products by the aged will differ in different places. We will perform further studies on aged groups in other provinces in the future.

Secondly, due to the various types of smart service products for the aged that are available, there may be some differences in the degree of use amongst the aged. In the future, research and data collection should be carried out in relation to different types of smart service products in order to build corresponding theoretical research models.

Lastly, aged users may face challenges of physical and cognitive decline, which may further affect their use of information technology. Therefore, future research should aim to explore the differences among aged groups in relation to physical condition and cognitive competence, and their impacts on the use of smart aged-care products.

## Figures and Tables

**Table 1:** Definitions of variable’s operability and the reference scales.

Category	Research Variables	Definition of Operability	Code	Measurement Item	Sources
UTAUT model	Performance expectations	The elderly believe that the use of smart aged-care products can help them acquire better services	PE1	I think smart aged-care products are easy to use and can promote the effect of elderly care	[21,58]
			PE2	The use of smart aged-care products can help me better enjoy elderly care service	
			PE3	I think the use of smart aged-care products can save time, and is convenient and fast	
			PE4	I think the use of innovative smart aged-care products and technology can promote the effect of elderly care	
			PE5	I think the use of smart aged-care products can promote the convenience of elderly care services	
	Effort expectancy	The ease of use of smart aged-care products considered by the elderly	EE1	I can easily learn to use smart aged-care products without spending too much time	[21,58]
			EE2	For me, the operation process of smart aged-care products is simple and easy	
			EE3	I fully understand and understand how to use smart aged-care products	
			EE4	The innovative application of smart aged-care products is not a challenge to me	
	Social influence	The extent to which the elderly are aware of whether others think they should use smart aged-care products	SI1	People around me influence my decision to use smart aged-care products	[21,58]
			SI2	People who use smart aged-care products look more capable than those who do not	
			SI3	The use of smart aged-care products is a trend. I want to keep up with the pace of the times, and I will use them	
			SI4	The use of smart aged-care products can improve personal image in society	
	Facilitating conditions	The extent to which the elderly believe that the existing supporting resources can support the use of smart aged-care products	FC1	I have the resources needed to use smart aged-care products	[21,58]
			FC2	I think smart aged-care products can match other technologies used	
			FC3	I have the necessary skills required to use smart aged-care products	
			FC4	When I encounter difficulties in using smart aged-care products, I can ask friends for help	
	Behavioral intention	The behavioral tendency of the elderly to use smart products	BI1	It’s a good idea to use smart aged-care products	[21,58]
			BI2	I think using smart aged-care products can promote my health	
			BI3	I think smart aged-care products are very valuable	
			BI4	I will use smart aged-care products in the future	
	Use behavior	The elderly use smart aged-care products	UB1	I am very willing to use smart aged-care products for health management	[21,58]
			UB2	I learn how to use smart aged-care products	
			UB3	Compared with other health care products, I prefer smart aged-care products	
			UB4	I will continue to use smart aged-care products	
Perceived cost theory	Perceived cost	The elderly think that using smart aged-care products involves more costs	PC1	Smart aged-care products are much more expensive than non-intelligent pension products	[31,59,60]
			PC2	I need to spend more time and energy on learning to use smart aged-care products	
			PC3	It takes more energy to use smart aged-care products	
Perceived risk theory	Perceived risk	The elderly think it is risky to use smart aged-care products	PR1	The use of smart aged-care products may cause financial losses	[61,62,63]
			PR2	The use of smart aged-care products may not meet my original expectations	
			PR3	The use of smart aged-care products makes me nervous or anxious	
			PR4	Use of smart aged-care products may cause harm to the body	
			PR5	The use of smart aged-care products may cause my information to be leaked	

**Table 2:** Questionnaire reliability analysis.

Variables	Item	Corrected Item–Total Correlation (CITC)	Cronbach’s α If Item Deleted	Cronbach’s α
Performance expectation	PE1	0.813	0.926	0.916
	PE2	0.848	0.92	
	PE3	0.833	0.923	
	PE4	0.835	0.922	
	PE5	0.83	0.923	
Effort expectation	EE1	0.822	0.916	0.909
	EE2	0.856	0.905	
	EE3	0.83	0.913	
	EE4	0.848	0.908	
Social influence	SI1	0.712	0.872	0.907
	SI2	0.751	0.857	
	SI3	0.758	0.855	
	SI4	0.797	0.839	
Facilitating conditions	FC1	0.78	0.851	0.906
	FC2	0.774	0.853	
	FC3	0.761	0.859	
	FC4	0.722	0.873	
Behavioral intention	BI1	0.857	0.908	0.898
	BI2	0.836	0.915	
	BI3	0.847	0.911	
	BI4	0.83	0.917	
Use behavior	UB1	0.756	0.859	0.895
	UB2	0.802	0.842	
	UB3	0.807	0.84	
	UB4	0.673	0.891	
Perceived cost	PC1	0.751	0.829	0.874
	PC2	0.768	0.814	
	PC3	0.756	0.825	
Perceived risk	PR1	0.885	0.952	0.935
	PR2	0.897	0.95	
	PR3	0.884	0.952	
	PR4	0.882	0.953	
	PR5	0.898	0.95	

**Table 3:** Basic information of respondents.

Item	Option	Frequency	Percentage (%)	Cumulative Percentage (%)
Gender	Male	187	48.45	48.45
	Female	199	51.55	100
Age	60–65 years old	65	16.84	16.84
	66–70 years old	97	25.13	41.97
	71–75 years old	108	27.98	69.95
	76–80 years old	88	22.80	92.75
	Over 80 years old	28	7.25	100
Education background	Senior high school and under	292	75.65	75.65
	Junior college	25	6.48	82.12
	Undergraduate	50	12.95	95.08
	Master’s and above	19	4.92	100
Disposable monthly income	Below CNY 2000	32	8.29	8.29
	CNY 2001–3500	78	20.21	28.50
	CNY 3501–5000	125	32.38	60.88
	Over CNY 5000	151	39.12	100
Total		386	100	100

**Table 4:** KMO and Bartlett’s test.

KMO Value		0.931
Bartlett test of sphericity	Chi-square approximation☐	9339.6
	df	528
	*p*-value	0

**Table 5:** Factor loadings (rotated).

Name	Factor Loading								Commonality (Common Factor Variance)
	Factor 1	Factor 2	Factor 3	Factor 4	Factor 5	Factor 6	Factor 7	Factor 8	
PE1	−0.105	**0.822**	0.129	0.135	0.083	0.127	0.12	−0.101	0.769
PE2	−0.071	**0.800**	0.157	0.125	0.119	0.146	0.146	−0.102	0.753
PE3	−0.099	**0.802**	0.095	0.111	0.136	0.180	0.128	−0.129	0.757
PE4	−0.100	**0.790**	0.162	0.173	0.106	0.078	0.162	−0.089	0.742
PE5	−0.092	**0.811**	0.097	0.077	0.122	0.18	0.107	−0.064	0.745
EE1	−0.114	0.147	0.177	0.179	**0.794**	0.191	0.126	−0.093	0.789
EE2	−0.107	0.136	0.102	0.153	**0.800**	0.204	0.126	−0.07	0.767
EE3	−0.155	0.155	0.121	0.121	**0.828**	0.100	0.131	−0.089	0.799
EE4	−0.112	0.111	0.189	0.176	**0.802**	0.203	0.15	−0.054	0.801
SI1	−0.127	0.173	0.096	**0.822**	0.143	0.135	0.197	−0.047	0.811
SI2	−0.057	0.115	0.122	**0.824**	0.144	0.145	0.102	−0.091	0.77
SI3	−0.069	0.152	0.098	**0.825**	0.154	0.111	0.097	−0.123	0.779
SI4	−0.135	0.137	0.138	**0.815**	0.147	0.136	0.113	−0.078	0.78
FC1	−0.050	0.131	**0.853**	0.109	0.123	0.049	0.109	−0.041	0.791
FC2	−0.025	0.140	**0.860**	0.100	0.112	0.097	0.103	−0.032	0.804
FC3	−0.004	0.148	**0.842**	0.093	0.087	0.04	0.074	−0.052	0.757
FC4	−0.006	0.119	**0.842**	0.111	0.172	0.06	0.085	−0.052	0.778
BI1	−0.215	0.154	0.137	0.135	0.159	0.237	**0.776**	−0.046	0.793
BI2	−0.186	0.181	0.077	0.117	0.202	0.192	**0.766**	−0.133	0.769
BI3	−0.241	0.216	0.150	0.183	0.115	0.198	**0.710**	−0.162	0.744
BI4	−0.211	0.201	0.140	0.177	0.126	0.229	**0.752**	−0.089	0.778
UB1	−0.088	0.15	0.094	0.125	0.163	**0.766**	0.25	−0.158	0.755
UB2	−0.093	0.215	0.083	0.145	0.177	**0.808**	0.118	−0.161	0.807
UB3	−0.086	0.199	0.031	0.164	0.259	**0.717**	0.279	−0.135	0.753
UB4	−0.085	0.225	0.088	0.194	0.19	**0.740**	0.211	−0.152	0.755
PC1	0.192	−0.107	−0.059	−0.075	−0.106	−0.138	−0.155	**0.831**	0.803
PC2	0.191	−0.147	−0.064	−0.139	−0.058	−0.187	−0.088	**0.825**	0.809
PC3	0.210	−0.166	−0.055	−0.104	−0.102	−0.16	−0.079	**0.816**	0.793
PR1	**0.846**	−0.106	0.021	−0.066	−0.107	−0.059	−0.198	0.088	0.793
PR2	**0.875**	−0.069	−0.015	−0.064	−0.086	−0.071	−0.12	0.125	0.818
PR3	**0.852**	−0.117	−0.014	−0.094	−0.092	−0.068	−0.102	0.144	0.793
PR4	**0.851**	−0.091	−0.072	−0.116	−0.118	−0.025	−0.135	0.110	0.795
PR5	**0.853**	−0.059	−0.021	−0.052	−0.067	−0.101	−0.13	0.147	0.788
Cumulative percentage of variance explained %									77.993

Note: Bold numbers are those for which factor loading is greater than 0.6.

**Table 6:** Summary table of confirmatory factor analysis.

Item			Estimate	S.E.	C.R.	*p*	Std	CR	AVE
PE1	<---	PE	1				0.839	0.916	0.686
PE2	<---	PE	1.022	0.052	19.818	***	0.835		
PE3	<---	PE	1.029	0.052	19.685	***	0.837		
PE4	<---	PE	1.003	0.052	19.19	***	0.819		
PE5	<---	PE	1.005	0.052	19.195	***	0.812		
EE1	<---	EE	1				0.855	0.909	0.713
EE2	<---	EE	0.953	0.048	19.686	***	0.821		
EE3	<---	EE	0.973	0.047	20.519	***	0.84		
EE4	<---	EE	1.028	0.049	20.934	***	0.862		
SI1	<---	SI	1				0.867	0.907	0.708
SI2	<---	SI	0.944	0.047	20.058	***	0.822		
SI3	<---	SI	0.939	0.046	20.36	***	0.834		
SI4	<---	SI	0.986	0.048	20.719	***	0.843		
FC1	<---	FC	1				0.851	0.907	0.708
FC2	<---	FC	1.097	0.052	20.918	***	0.866		
FC3	<---	FC	0.966	0.051	19.062	***	0.807		
FC4	<---	FC	1.052	0.052	20.092	***	0.842		
BI1	<---	BI	0.999	0.05	19.941	***	0.845	0.898	0.688
BI2	<---	BI	0.966	0.051	18.921	***	0.816		
BI3	<---	BI	0.955	0.051	18.869	***	0.817		
BI4	<---	BI	1				0.84		
UB1	<---	UB	0.98	0.054	18.093	***	0.808	0.874	0.699
UB2	<---	UB	1.063	0.055	19.284	***	0.832		
UB3	<---	UB	1.036	0.055	18.862	***	0.831		
UB4	<---	UB	1				0.831		
PC1	<---	PC	1.003	0.056	17.887	***	0.823	0.895	0.681
PC2	<---	PC	1.069	0.058	18.418	***	0.85		
PC3	<---	PC	1				0.835		
PR1	<---	PR	1				0.857	0.935	0.742
PR2	<---	PR	1.021	0.045	22.831	***	0.879		
PR3	<---	PR	0.998	0.045	21.947	***	0.859		
PR4	<---	PR	1.013	0.046	22.098	***	0.858		
PR5	<---	PR	0.972	0.045	21.752	***	0.853		

Note: *** *p* < 0.001.

**Table 7:** Discriminant validity of the measurement model.

	AVE	PE	EE	SI	FC	PR	BI	UB	PC
PE	0.686	**0.828**							
EE	0.713	0.430 ***	**0.845**						
SI	0.708	0.436 ***	0.483 ***	**0.842**					
FC	0.708	0.388 ***	0.409 ***	0.345 ***	**0.842**				
PR	0.742	−0.299 ***	−0.338 ***	−0.296 ***	−0.124 *	**0.861**			
BI	0.681	0.541 ***	0.582 ***	0.496 ***	0.297 ***	−0.312 ***	**0.825**		
UB	0.688	0.527 ***	0.516 ***	0.495 ***	0.373 ***	−0.498 ***	0.667 ***	**0.830**	
PC	0.699	−0.405 ***	−0.346 ***	−0.357 ***	−0.215 ***	0.451 ***	−0.513 ***	−0.440 ***	**0.836**

Note: The square root of AVE (shown as bold at diagonal).* *p* < 0.05, *** *p* < 0.001.

**Table 8:** Model fit index.

Model Fit	Criteria	Model Fit of Research Model	Judgment
ML chi-square (χ2)	The smaller the better	519.53	
Degrees of Freedom (df)	The larger the better	472	
Normed Chi-square (χ2/df)	<3	1.101	Yes
Root Mean Square Error Approximation (RMSEA)	<0.08	0.037	Yes
Standardized Root Mean Square Residual (SRMR)	<0.08	0.0461	Yes
Tucker–Lewis Index (TLI)	>0.9	0.946	Yes
Comparative Fit Index (CFI)	>0.9	0.951	Yes
Goodness of Fit Index (GFI)	>0.9	0.918	Yes
Incremental Fit Index (IFI)	>0.9	0.954	Yes

**Table 9:** Verification results of hypotheses.

Hypothesis	Route			Estimate	S.E.	C.R.	*p*	STD	Results
H1	BI	<---	PE	0.274	0.051	5.416	***	0.277	Support
H2	BI	<---	EE	0.27	0.056	4.805	***	0.272	Support
H3	BI	<---	SI	0.188	0.049	3.845	***	0.199	Support
H4	UB	<---	FC	0.017	0.055	0.304	0.761	0.018	Nonsupport
H5	UB	<---	BI	0.823	0.099	8.29	***	0.860	Support
H6	BI	<---	PC	−0.254	0.054	−4.69	***	−0.251	Support
H7	BI	<---	PR	−0.135	0.058	−2.334	0.02	−0.152	Support
H8	UB	<---	PR	0.082	0.067	1.221	0.222	0.096	Nonsupport

Note: *** *p* < 0.001.

## Data Availability

Not applicable.
